# Uncertainty in NIST Force Measurements

**DOI:** 10.6028/jres.110.084

**Published:** 2005-12-01

**Authors:** Tom Bartel

**Affiliations:** National Institute of Standards and Technology, Gaithersburg, MD 20899-8222

**Keywords:** calibration, deadweight force standard, expanded uncertainty, force, gravity, mass, transfer standard, metrology, uncertainty

## Abstract

This paper focuses upon the uncertainty of force calibration measurements at the National Institute of Standards and Technology (NIST). The uncertainty of the realization of force for the national deadweight force standards at NIST is discussed, as well as the uncertainties associated with NIST’s voltage-ratio measuring instruments and with the characteristics of transducers being calibrated. The combined uncertainty is related to the uncertainty of dissemination for force transfer standards sent to NIST for calibration.

## 1. Introduction

For more than 75 years NIST has maintained a force laboratory capable of disseminating force measurement standards to government, industry, and academic facilities through the calibration of force transducers that serve as transfer standards. The facilities available at NIST, the services provided, and the procedures employed have been described in previous publications [1–5]. The purpose of this paper is to develop an uncertainty estimate for NIST force measurements, based on an examination of the various uncertainty contributors that apply to the present primary force standard facilities.

The NIST primary force standards consist of six machines for applying discrete forces generated by stainless steel deadweights, spanning a range of 44 N to 4.448 MN [1]. These machines were constructed about the year 1965, becoming operational following the completion of the deadweight mass determinations in 1966. Automation of the weight-changing mechanisms of these machines was accomplished about 1989, along with the implementation of instruments for the precise automated measurement of the responses of strain gauge load cells used for measuring force.

Section 2 of this paper presents the form of the transducer calibration equation as a framework for proceeding with the examination of various force uncertainty components. Following that are discussions of the uncertainties associated with the realization of force (Sec. 3), the measurement of transducer response (Sec. 4), and the fit of the data to the calibration equation (Sec. 5). It is noted that the uncertainty components of Sec. 5, which are largely dependent upon the characteristics of the transducer being calibrated, are the dominant contributors of the overall measurement uncertainty.

## 2. Expression of Uncertainty for the Force Calibration Equation

Current force transducer designs do not incorporate an absolute internal reference for the measure of force. Rather, a force transducer can achieve an accuracy of 0.01 % or better only through calibration relative to a known reference. To be fully useful, a transducer must be accompanied by its particular calibration equation relating the transducer output response to the applied force.

A force transducer’s response is generally expressed in terms of the applied force by a polynomial equation:

$$R=A_{0}+\Sigma A_{i}F^{i},$$ (1)

where *R* is the transducer response, *F* is the applied force, and the *A_i_* are coefficients characterizing the transducer. In practice, the summation is usually carried to an order of 2 or 3. The unit for *R* is appropriate for the type of deflection-sensing system employed by the transducer, which may be mechanical (in proving rings, for example), electronic (for strain gauge load cells), or hydraulic.

NIST provides a force calibration service whereby the response *R_j_* of a customer’s transducer is measured for each of several applied reference forces *F_j_*, with the forces applied in a sequence in accordance with an appropriate test method such as ASTM E 74-04 [6]. The coefficients *A_i_* in Eq. (1) are then calculated from a least-squares fit to the data set (*F_j_*, *R_j_*).

Thus the “disseminated result” of a force calibration at NIST is the set of coefficients *A_i_* for the particular transducer being calibrated. The uncertainty in this disseminated result is attributable to the uncertainty in the applied forces, the uncertainty in the calibration of the instrumentation used to acquire the transducer responses, and the uncertainty of the fit of the measured data to the equation chosen as a model, which can be attributed in part to certain characteristics of the transducer. These quantities are denoted as *u*_f_, *u*_v_, and *u*_r_ in Eq. (2).

For each NIST force calibration report, this measurement uncertainty is given as the expanded uncertainty, *U*, which is calculated in accordance with NIST Technical Note 1297, “Guidelines for Evaluating and Expressing the Uncertainty of NIST Measurement Results” [7]. The NIST policy stated in this document is based on an approach presented in detail by the ISO publication, “Guide to the Expression of Uncertainty in Measurement,” ISBN 92-67-10188-9 (1993) [8].

The expanded uncertainty *U* is reported in units of the transducer response, providing the uncertainty in the response values calculated from the calibration equation yielded by the NIST calibration measurements. Thus *U* defines an interval *R* ± *U*, within which the response of the transducer to a given applied force is expected to lie, when *R* is calculated from the calibration coefficients *A_i_* according to Eq. (1).

The value of *U* is calculated by multiplying the combined standard uncertainty, *u*_c_, by a coverage factor, *k*, of 2. Thus the confidence level for the interval defined above is about 95 %.

The combined standard uncertainty, *u*_c_, is determined from

$${u_{c}}^{2}={u_{f}}^{2}+{u_{v}}^{2}+{u_{r}}^{2},$$ (2)

where:

*u*_f_ is the standard uncertainty associated with the applied force, due to uncertainties in the mass calibration and adjustment of the dead weights and to uncertainties in the air density and the acceleration of gravity. This component is explained in Sec. 3.
*u*_v_ is the standard uncertainty in the calibration of the voltage ratio measurement instrumentation used at NIST. This component does not apply if the force transducer being calibrated incorporates an indicating instrument that is part of the calibrated device. This component is explained in Sec. 4.
*u*_r_ is the standard deviation calculated according to ASTM E 74-04 from the differences between the individual measured responses and the corresponding responses computed from Eq. (1). An explanation of this calculation is given in Sec. 5.

## 3. Uncertainty in the Applied Force

The NIST deadweight force standards exert force by means of the earth’s gravitational attraction acting upon weights of calibrated mass. The downward force exerted on a static deadweight is given by

$$F=mg\left[1-\left(\rho_{a}/\rho_{w}\right)\right],$$ (3)

where *F* is the applied force in *N*, *m* is the mass of the weight in kg, *g* is the acceleration of gravity in m/s^2^, *ρ*_a_ is the atmospheric density at the location of the weight, and *ρ*_w_ is the density of the weight in the same units as *ρ*_a_. The uncertainty in this force is dependent upon the uncertainties in the measured values of the mass, gravitational acceleration, and the ratio of the air and weight densities, which are discussed respectively in Secs. 3.1, 3.2, and 3.3.

Uncertainties associated with transducer mounting in the force machine, such as the placement of the point of force application on the transducer or the alignment of the vertical gravity vector with the load cell axis, are discussed in Sec. 5.

### 3.1 Uncertainty Associated with Mass

All of the weights for each of the six NIST deadweight machines had their masses determined in 1965 and 1966 by the mass laboratory at NIST, which was called the National Bureau of Standards prior to 1988. The organizational name for the mass laboratory at the time was the Institute for Basic Standards, Metrology Division, Mass and Volume Section, with Paul E. Pontius serving as the section chief. Mass and force metrologies are currently organized at NIST within one group under the Manufacturing Engineering Laboratory, Manufacturing Metrology Division, Mass and Force Group [1].

The deadweight masses were determined by comparisons with U.S. national mass standards, with the procedure also incorporating adjustments of the weights to achieve the desired mass values. The reports of calibration giving the results of the mass determinations performed in 1965 and 1966 provide the uncertainty for each mass as a standard deviation representing “a limit to the effect of random errors of measurement plus systematic error from known sources.” Those analyses thus incorporate all known Type A and Type B uncertainty components. The reported values yield standard uncertainties for the individual deadweight masses that range from 0.0001 % to 0.0003 % of the mass values.

Since the masses of the individual weights of each machine were determined similarly, the mass values may be partially correlated; thus the combined mass uncertainty of any combination of masses may more appropriately be taken to be the sum of the individual uncertainties rather than the square root of their estimated variance. This combined uncertainty will then lie in the range from 0.0001 % to 0.0003 % of the mass of the combination. Rather than compute separate combined uncertainties for different combinations of weights, the upper end of the range, 0.0003 %, for the relative standard uncertainty of the individual masses is regarded to represent a reasonable value for the relative standard uncertainty for any combination of masses.

Thus the standard uncertainty in the applied force that is associated with the uncertainty in the determination of the deadweight masses is no greater than 0.0003 % of the applied force. The combined standard uncertainty given in Eq. (2) is expressed in transducer response units; thus the standard uncertainty in the applied force must be transformed into equivalent transducer response units. Since the determined response *R* given in Eq. (1) is approximately a linear function of the applied force *F*, the standard uncertainty, *u*_fa_, in the response *R* that is associated with the uncertainty in the determination of the deadweight masses is no greater than 0.0003 % of the transducer response *R*. Thus

$$u_{\text{fa}}\leq 0.000003\;R.$$ (4)

This value represents an upper bound to the relative standard uncertainty for any combination of weights.

The question of whether the deadweight masses change with time must be addressed. Possible mechanisms for such mass change are the outgassing of entrapped gases from the deadweight material, the occurrence of oxidation or other chemical activity, or the adsorption of contaminants. To minimize the possibility of such variation in the deadweight masses with time, the weights were made of stainless steel. For the 498 kN, 1.334 MN, and 4.448 MN machines, the American Iron and Steel Institute (AISI) series 410 alloy was chosen because of its superior strength and resistance to galling at the weight-bearing contact surfaces. The design of the 2.2 kN, 27 kN, and 113 kN machines, incorporating independent loading mechanisms for each weight, minimizes the possibility for galling; thus the 300 series alloy was chosen for these machines.

**Fig. 1:**
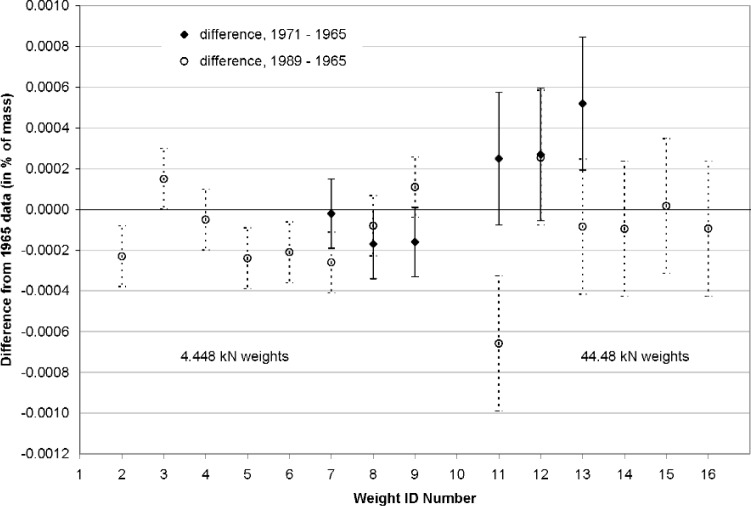
Comparison of mass values determined in 1965, 1971, and 1989 for the NIST 498 kN deadweight machine.

The 498 kN machine was partially disassembled for service in 1971 and again in 1989, providing opportunities for observing whether significant mass changes in its weights were taking place. New mass determination measurements were conducted in each of those years for the weights that were removed. These weights were organized into two sets, with individual weights of each set yielding forces of 4.448 kN and 44.48 kN, respectively. A comparison of the masses for these weights for the 1965, 1971, and 1989 determinations is shown in Fig. 1. The points on this plot that are depicted with solid symbols represent the differences between the 1971 and 1965 mass values, given in percent of each respective mass. Positive values represent an apparent increase in mass since 1965. The corresponding uncertainty intervals represent the combined standard uncertainties for the 1971 and 1965 mass determinations, given in percent of each respective mass. The points on the plot that are depicted with open symbols represent the relative mass differences, along with the combined standard uncertainties, for the 1989 and 1965 measurements.

All but two of the points in Fig. 1 lie within ±0.0003 %, which is the upper bound value for the relative standard uncertainty in the determination of the mass. The individual standard uncertainty intervals depicted by the error bars, having a confidence level of approximately 68 %, are seen to enclose the baseline for fourteen of the twenty points. None of the deviations exceed their respective expanded uncertainties, for which the confidence level is approximately 95 %. The mean difference for the twenty points is −0.0001 %, which is not sufficient to establish a significant systematic mass change phenomenon from these observations.

In order to more completely address the question of stability of NIST’s deadweight masses, the 2.2 kN machine was completely disassembled in 1996 and new mass determination measurements were performed for all of its weights. The 2.2 kN machine was selected because it has the smallest weights, which provide the largest ratio of surface area to mass. Under the assumption that any long term mass change involves a surface effect, the relative change would be greater, and thus more observable, for the smaller weights. In addition, this effort enabled a check on the alloy used for the three smaller machines.

**Fig. 2:**
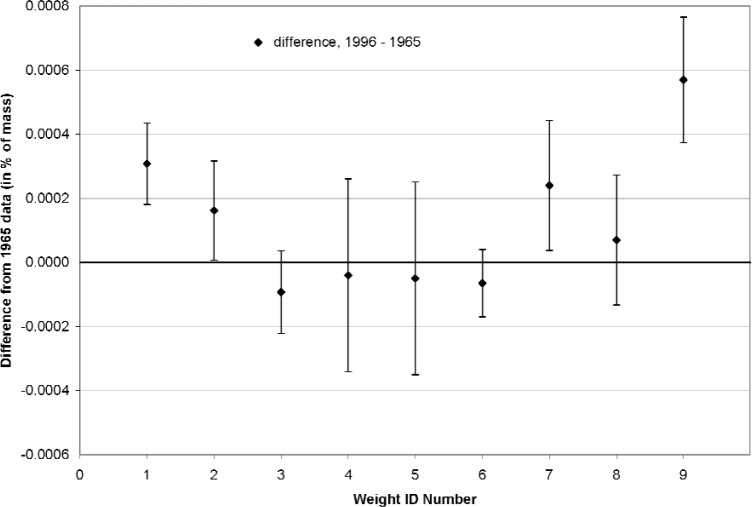
Comparison of mass values determined in 1965 and 1996 for the NIST 2.2 kN deadweight machine.

A comparison of the 2.2 kN machine masses for the 1965 and 1996 determinations is shown in Fig. 2. The points represent the differences between the 1996 and 1965 mass values, given in percent of the each respective mass. As in Fig. 1, positive values represent an apparent increase in mass since 1965. The error bars represent the combined standard uncertainties for the 1996 and 1965 mass determinations, given in percent of each respective mass. The uncertainty invervals differ in length because the mass uncertainty for each weight is calculated from the data for that weight.

One point in Fig. 2 lies outside ±0.0003 %, which is the upper bound value for the standard uncertainty in the determination of the mass. The individual standard uncertainty intervals are seen to lie outside of the baseline for four of the nine points. While two of the deviations exceed their respective expanded uncertainties for a coverage factor of two, the mean difference of +0.0001 % is not sufficient to establish a significant systematic mass change phenomenon from these observations. Since the larger NIST deadweight machines would incur smaller relative mass changes than the 2.2 kN machine, it is concluded that significant changes in deadweight mass are not evident in the NIST force laboratory facilities.

A diligent quality assurance program of inspections, maintenance, and security serves to provide confidence that mass changes in the weights are not occurring through contamination, fluid leakage, extraneous objects, or mechanical wear.

### 3.2 Uncertainty Associated with Gravitational Acceleration

The absolute value of the acceleration due to gravity, denoted as g in Eq. (3), was determined in 1965 at the NIST force laboratory in Gaithersburg, MD by means of free-fall measurement apparatus constructed by Doug Tate [9]. The equipment consisted of a 1 m long fused silica tube that was allowed to fall freely within a vacuum chamber; this vacuum chamber itself was allowed to fall under the influence of gravity, restrained only by guide rods involving minimal friction. The position of the falling silica tube as a function of time was determined by means of slits cut into the tube at carefully measured positions, allowing light to pass from an external light source horizontally through the tube. Transparent ports located in the falling vacuum chamber allowed detection by an external light sensor when any of the falling slits aligned momentarily with a stationary reference slit. The total height of the free fall was about 1.25 m.

D. R. Tate’s instrumentation enabled the gravitational acceleration to be established for a reference point within the force laboratory, giving a value for *g* of 9.801018 m/s^2^. This value is 0.0574 % less than the nominal sea level value for *g* of 9.806650 m/s^2^. Tate stated a measurement standard deviation of 0.000005 m/s^2^, which is about 0.00005 % of the measurement result. The measurement procedure also allowed a determination of the gravity gradient, which was −0.000003 s^−2^. This measured value for the gravity gradient is about the same as that which can be calculated from Newton’s gravitational equation if the earth were assumed to be a sphere of radius *R*_e_, having a mass *M*_e_ of spherically symmetric distribution. At a distance *r* from the earth’s center, where *r* ≥ *R*_e_, the gravitational acceleration would be

$$g=GM_{e}/r^{2},$$ (5)

where *G* is the Newtonian gravitational constant. The value for *g* at the earth’s surface is *g*_s_ = *GM*_e_/*R*_e_^2^. Over a small differential Δ*r* in height at the earth’s surface, the gravity gradient can be computed from Eq. (5) as

$$\Delta g/\Delta r\approx -2g_{s}/R_{e}.$$ (6)

Taking the earth radius as 6.379 × 10^6^ m, Eq. (6) yields Δ*g*/Δ*r* ≈ −0.000003 s^−2^, about the same as observed by Tate.

New determinations of the gravitational acceleration were obtained from a gravity survey of the NIST force laboratory in 1992, performed by the National Oceanic and Atmospheric Administration (NOAA), Office of Ocean and Earth Sciences, Gravity Section. This survey was performed with portable equipment brought by NOAA personnel; the equipment employed an automated short distance free-fall mechanism, sensed with laser interferometry, that could be operated repeatedly over a period of time. This survey yielded gravity values at six locations throughout the laboratory. At the reference point characterized by Tate, the NOAA value for *g* was 9.80101353 m/s^2^ ± 0.00000008 m/s^2^; this is smaller than Tate’s value by about 0.000004 m/s^2^, which is about the same as the standard uncertainty in Tate’s measurements. Thus the difference between the Tate and NOAA measurements is within the expanded uncertainty interval.

The NIST deadweight masses were determined in 1965 and 1966, following Tate’s gravity measurements. In preparation for this analysis, the value for *g* at the midpoint of each of the six NIST deadweight stacks was derived from the absolute gravitational acceleration at Tate’s reference location and the gravity gradient. During the mass determination, each weight was adjusted to exert its nominal force for the value of *g* at its stack’s midpoint. The only significant uncertainty associated with *g* in Eq. (3) is the variation of *g* with height relative to the midpoint of each weight stack. The largest height variation relative to the stack midpoint in the NIST force laboratory is 5.5 m. Rather than make individual corrections for the location of each weight, an associated uncertainty is estimated on the basis of a rectangular probability distribution as described in Sec. 4.6 of NIST Technical Note 1297, “Guidelines for Evaluating and Expressing the Uncertainty of NIST Measurement Results” [7]. The corresponding relative standard uncertainty in *g*, and thus in *F*, is given by the (largest height variation) × (relative gravity gradient) × 3^−0.5^, or 0.000001. Combining this uncertainty with the uncertainty in Tate’s absolute gravity measurements yields a standard uncertainty in the applied force *F*, associated with the uncertainty in the gravitational acceleration, of about 0.000001 *F*. The corresponding standard uncertainty, *u*_fb_, in the response *R*, resulting from the uncertainty in the gravitational acceleration, is given by

$$u_{\text{fb}}\approx 0.000001\;R.$$ (7)

This uncertainty component could be eliminated through computation, by calculating *g* from the measured height for each weight. Computation of the equivalent height of the weight frame of each machine would require an integration over the distributed mass of the frame, which constitutes the first calibrated weight of the weight stack.

A discussion of the current state of the art in the measurement of gravitational acceleration is given by J. E. Faller [10], of the Quantum Physics Division of the NIST Physics Laboratory in Boulder, CO. An online model enabling the prediction of surface gravity for any point within the continental United States can be accessed from the tools section of the Internet web site of the National Geodetic Survey (NGS), which has the address www.ngs.noaa.gov. The predictions are calculated by interpolation from observed gravity data contained in the National Spatial Reference System of the NGS. The uncertainty in the interpolation, which is provided for each location specified by the requester, has relative values that are typically about 0.000005. Application of this online model to the location of the reference point in the NIST force laboratory characterized by Tate yields a value of 9.80102 m/s^2^ ± 0.00002 m/s^2^, which is consistent with the fact that past gravimetric measurements at NIST contribute to the NGS database.

### 3.3 Uncertainty Associated with Density

The adjustment of the weights in 1965 and 1966, which incorporated the local value of *g* as described above, also incorporated the average local value for the buoyancy factor [1 − (*ρ*_a_/*ρ*_w_)] that appears in Eq. (3). The value of *ρ*_a_ used in these adjustments was the year-round mean air density in Gaithersburg, MD, of 1.17 kg/m^3^ as discussed below. The density *ρ*_w_ of the stainless steel material of the weights was determined by associates of Doug Tate at NIST, by determining the mass for small cylindrical specimens of the material for which the volume was also determined from dimensional measurements. The values obtained were 7720 kg/m^3^ for the AISI 410 alloy, used for the three larger deadweight machines, and 7890 kg/m^3^ for the AISI 300 alloy of the three smaller machines. The standard uncertainty in these measurements was less than 1 %. The application of the buoyancy correction involves a relative reduction, *ρ*_a_/*ρ*_w_, in the applied force of 0.0152 % for AISI 410, and 0.0148 % for AISI 300.

Without corrections made to the applied force for daily fluctuations in air density at NIST, the uncertainty associated with the use of the mean air density must incorporate these normal weather related fluctuations. Paul Pontius [11] provides a compilation of the average air densities, derived from Weather Bureau data, for selected cities throughout the continental United States. The average air density for Washington, DC, corrected for a constant temperature of 23 °C, is given as 1.185 kg/m^3^ ± 0.04 kg/m^3^, where the limits define the range over which the actual air density may fluctuate through the year. The difference in elevation between Gaithersburg, MD and Washington, DC is approximately 120 m. According to documentation published jointly by the National Oceanic and Atmospheric Administration, the National Aeronautics and Space Administration, and the U.S. Air Force [12], this difference in elevation reduces the air pressure, and thus the air density, by about 1.4 %. Employing this correction yields the 1.17 kg/m^3^ mean air density used for the weight adjustments; the range of actual air density fluctuation remains as ± 0.04 kg/m^3^, giving an interval of 1.13 kg/m^3^ to 1.21 kg/m^3^.

The variation of ± 0.04 kg/m^3^ in *ρ*_a_ corresponds to a relative change in the applied force of ± 0.0005 %, computed from Eq. (3) and using the density of either alloy for *ρ*_w_. An associated uncertainty is estimated on the basis of a rectangular probability distribution, giving an estimated relative standard uncertainty of 0.000005 × 3^−0.5^. Thus the standard uncertainty in the applied force *F*, resulting from the variation of actual air density from the yearly mean air density, is about 0.0003 *F*. The corresponding standard uncertainty, *u*_fc_, in the response *R*, resulting from the variation of actual air density from the yearly mean air density, is given by

$$u_{\text{fc}}\approx 0.000003\;R.$$ (8)

This uncertainty component could also be eliminated through computation, provided that the barometric pressure, humidity, and temperature are sampled throughout each force calibration.

The NIST Mass and Force Group has some accumulated barometric pressure data that can corroborate the air density interval used in the above uncertainty calculation. For the past eleven years NIST has been performing legal metrology load cell evaluations in accordance to specifications given by the Organization Internationale de Métrologie Légale (OIML) [13] and by the National Type Evaluation Program (NTEP) [14]. Discussions of NIST’s conduct of these procedures have been given previously [15]. During these measurements, the barometric pressure is recorded continuously, typically at 5 min intervals, for a period of two or more days for each load cell evaluation.

The barometric pressure data from legal metrology evaluations using the NIST 498 kN deadweight machine have been extracted for a 5 year period beginning in 1998. These measurements involve evaluations on forty load cells, spaced somewhat randomly over the 5 year period, and incorporating a total accumulated measurement time of 90 days. Of the 25 000 individual barometric pressure samples taken in these measurements, the average, minimum, and maximum values are 100.13 kPa, 98.15 kPa, and 102.33 kPa, respectively.

The air density *ρ*_a_ can be calculated from the barometric pressure if other atmospheric parameters are also known, using an internationally accepted equation [16] of the form

$$\rho_{a}=\left(pM_{a}/ZR_{g}T\right)\left[1-x_{v}\left(1-M_{v}/M_{a}\right)\right],$$ (9)

where *p* is the atmospheric pressure, *T* is the thermodynamic temperature, *x*_v_ is the mole fraction of water vapor, *M*_a_ is the molar mass of dry air, *M*_v_ is the molar mass of water, *R*_g_ is the molar gas constant, and *Z* is the compressibility factor. Necessary constants and supplementary relations are given in Ref. [16].

The temperature in the NIST force laboratory is regulated to 23 °C ± 0.2 °C in the rooms where the load cells are loaded, and to 23 °C ± 2 °C in the rooms housing the deadweights. In addition, the relative humidity typically ranges from 10 % to 60 %. Using Eq. (9) with the extremes of the ranges for the barometric pressure, air temperature, and relative humidity as given above, the average, minimum, and maximum values for the air density in the vicinity of the NIST deadweights are obtained as 1.17 kg/m^3^, 1.14 kg/m^3^, and 1.21 kg/m^3^, respectively. These results are essentially identical to the air density values derived from the Weather Bureau data given in Ref. [11]. Thus Eq. (8) remains as an adequate estimator for the uncertainty in the force associated with the air density.

The uncertainty in the applied force that is associated with the material density of the weights is now to be discussed. As indicated above, measurements at NIST of the density, *ρ*_w_, of the stainless steel material of the weights was believed to be accurate to about one percent. The problem is to determine what error in *F* is caused by an error in the value of *ρ*_w_.

This problem can be addressed by noting that the 1965 mass determinations were performed at NIST in air at ambient atmospheric pressure, with the temperature and humidity controlled to the same values as stated above. The mass determinations did not involve separate density measurements; instead, they used as input the same values for *ρ*_w_ that were determined by associates of Doug Tate as described above and used in Eq. (3) for all subsequent force measurements employing these weights. Thus for any weight, the value of the mass *m* of the weight is related to *ρ*_w_ by

$$mg\left[1-\left(\rho_{a}/\rho_{w}\right)\right]=m_{s}g\left[1-\left(\rho_{a}/\rho_{s}\right)\right],$$ (10)

where *m*_s_ is the mass of the mass standard used to determine *m*, and *ρ*_s_ is the density of this mass standard. This relation assumes a simplified case of a single mass standard and a gas density equal to the mean air density at NIST. Thus

$$m=m_{s}\left[1-\left(\rho_{a}/\rho_{s}\right)\right]/\left[1-\left(\rho_{a}/\rho_{w}\right)\right].$$ (11)

This mass value *m* is subsequently used in the force laboratory to determine the applied force, *F*, using Eq. (3). Since the uncertainty in *F* caused by a change in air density between the time of mass determination and the time of force application has already been accounted for, the same value for *ρ*_a_ may be used in both Eq. (3) and Eq. (11).

Suppose, however, that it is later discovered that the value *ρ*_w_ has significant error, and that the true value for the density of the weight is ${\rho_{W}}^{\prime }$.

The question is: what is the corresponding error in the force; i.e., what is the true force *F*′ corresponding to the true density ${\rho_{W}}^{\prime }$? In order to answer that question, one must first ask: what is the true mass *m*′ based on the mass determination performed earlier?

$$\begin{array}{l} m^{\prime }=m_{s}\left[1-\left(\rho_{a}/\rho_{s}\right)\right]/\left[1-\left(\rho_{a}/{\rho^{\prime }}_{w}\right)\right]\\ m^{\prime }=m_{s}\left\{\left[1-\left(\rho_{a}/\rho_{s}\right)\right]/\left[1-\left(\rho_{a}/\rho_{w}\right)\right]\right\} \end{array}$$ (12)

$$\cdot \left\{\left[1-\left(\rho_{a}/\rho_{w}\right)\right]/\left[1-\left(\rho_{a}/{\rho^{\prime }}_{w}\right)\right]\right\}$$ (13)

$$m^{\prime }=m\left[1-\left(\rho_{a}/\rho_{w}\right)\right]/\left[1-\left(\rho_{a}/{\rho^{\prime }}_{w}\right)\right].$$ (14)

With the mass so corrected, the correct force may now be calculated as

$$F^{\prime }=m^{\prime }g\left[1-\left(\rho_{a}/{\rho^{\prime }}_{w}\right)\right]$$ (15)

$$F^{\prime }=mg\left\{\left[1-\left(\rho_{a}/\rho_{w}\right)\right]/\left[1-\left(\rho_{a}/{\rho^{\prime }}_{w}\right)\right]\right\}\cdot \left[1-\left(\rho_{a}/{\rho^{\prime }}_{w}\right)\right]$$ (16)

$$F^{\prime }=mg\left[1-\left(\rho_{a}/\rho_{w}\right)\right]$$ (17)

$$F^{\prime }=F.$$ (18)

Thus the answer is: there is no error in *F* caused by an error in *ρ*_w_.

### 3.4 Standard Uncertainty Associated with the Applied Force

The standard uncertainty, *u*_f_, in the transducer response, incorporating all significant uncertainty components in the applied force, may now be calculated from

$${u_{f}}^{2}={u_{\text{fa}}}^{2}+{u_{\text{fb}}}^{2}+{u_{\text{fc}}}^{2},$$ (19)

where *u*_fa_, *u*_fb_, and *u*_fc_ are given by Eqs. (4), (7), and (8), respectively. For the forces applied by the NIST deadweight machines, this calculation yields

$$u_{f}=0.000005\;R.$$ (20)

## 4. Uncertainty in the Calibration of NIST Voltage-Ratio Instrumentation

As discussed under Eq. (1), each force *F*_j_ applied to a transducer undergoing force calibration is paired with a response *R*_j_ of the transducer to that applied force. The uncertainty in acquiring each response datum *R*_j_ results from the following two sources: (a) a “random” component related to the resolution of the transducer response indicating device and any variation in the responses such as would be seen in successive readings of the indicating device for a constant force input; and (b) a “systematic” component related to the calibration of the instrumentation used to acquire the responses.

The uncertainties identified by item (a) contribute to the deviations in the responses from the least-squares fit to the data and are accounted for by the uncertainty *u*_r_ discussed below in Sec. 5. The uncertainties of item (b) apply only if the responses are acquired by an indicating device that is not considered to be integrated with the force transducer being calibrated.

Many transducers calibrated at the NIST force laboratory are combined with indicating systems that are not separated from the transducers. Typical examples are mechanical systems, such as the micrometer screw and precisely machined contact points that are integrated within proving ring transducers, and electrical voltage-ratio measuring instruments supplied by customers for connection to strain gauge load cells. If an indicating instrument accompanies a transducer and is used by the customer in the same manner, without readjustment, as employed during calibration, then the indicating instrument is considered to be part of the calibrated system. Any systematic characteristics of the instrument are then accounted for by the calibration relation returned in the form of Eq. (1) by the procedure.

The NIST force laboratory maintains its own strain gauge excitation and voltage-ratio measuring instruments for use in calibrating load cells that are not accompanied by customer supplied indicating instruments. Because the calibration of NIST’s equipment is not integrated with the transducer calibration, the NIST Mass and Force Group must maintain a separate calibration of this instrumentation relative to national voltage standards. The uncertainty *u*_v_ of this electrical calibration must be incorporated into the combined standard uncertainty *u*_c_ of the force calibration procedure.

The NIST indicating system supplies direct current excitation to the load cell through the use of a DC power supply, which applies voltages to the load cell excitation input leads of ±5 V relative to the load cell ground wire, thus giving 10 V between the leads. This 10 V difference, serving as the excitation voltage, is stable to within ±5 µV over a time period of 15 s. This power supply was designed to internally switch the wires going to the ±5 V terminals by means of a computer command, thus reversing the polarity of the excitation signal to the load cell. This action makes it possible to cancel out small thermal biases in the strain gage bridge and connecting wires, as well as any zero-offsets in the rest of the indicating system. The switching is not done if the load cell is not designed to accommodate reversed polarity excitation.

The NIST indicating system simultaneously samples the excitation voltage and the load cell output voltage with an 8½ digit computing multimeter operating in voltage-ratio mode; the multimeter calculates the corresponding voltage ratio internally and returns that value in digital form to the computer. The multimeter is read twice, with the excitation voltage polarity reversed between readings; the final voltage ratio is taken as the average of the voltage ratios measured at each polarity. The meter sampling time at each polarity, and the delay after switching polarity before resuming the sampling, are specified by the operator through the computer control/acquisition program. A typical time for one complete voltage ratio reading is 10 s; this time can be shortened or lengthened as appropriate for the measurement being conducted.

### 4.1 Calibration Relative to NIST Voltage Standards

Use of NIST instrumentation to obtain the load cell responses during force calibrations mandates that the voltage ratio measurements be traceable to U.S. national electrical standards. This is accomplished by periodic “primary” calibration of the force laboratory’s computing multimeters by the Quantum Electrical Metrology Division of the NIST Electronics and Electrical Engineering Laboratory. This procedure is carried out in the multimeter’s voltage-ratio mode, by providing direct current voltage signals simultaneously to both input channels, with the calibrated signals derived from 1 V and 10 V Josephson-junction array voltage standards (JVS) maintained by the Quantum Electrical Metrology Division [17,18]. Such a calibration is performed by that division on at least one of the force laboratory multimeters per year. Different multimeters are selected for succeeding calibrations in order to avoid bias that could be associated with the calibration of the same meter repeatedly. The Mass and Force Group maintains calibration of all of its multimeters at least quarterly by comparison with the multimeters most recently calibrated by the Quantum Electrical Metrology Division, as described in the next section.

During the multimeter voltage-ratio calibration the Quantum Electrical Metrology Division maintains a 10 V signal from a solid-state dc voltage standard calibrated against the 10 V JVS at the meter’s ratio reference input, while applying a sequence of reference signals ranging from 5 mV to 100 mV provided by the 1 V JVS to the meter’s primary input channel. The corresponding voltage-ratio range is from 0.5 mV/V to 10 mV/V. For most load cells calibrated at NIST, the output when loaded to capacity is 2 mV/V to 4 mV/V.

The reference voltages derived from the Josephson voltage standard system are known with uncertainties of about 0.05 µV, provided that the sampling time of the multimeter is not greater than 10 s. This uncertainty corresponds to 0.00005 % of a 10 mV/V meter reading, or 0.0002 % of a multimeter reading of 2.5 mV/V. For a 10 s sampling time, the standard uncertainty in the multimeter voltage-ratio readings is 0.00001 mV/V, corresponding to 0.0001 % at 10 mV/V, or 0.0004 % at 2.5 mV/V.

From the Quantum Electrical Metrology Division measurements a meter calibration factor may be calculated, taken as the quotient of the voltage-ratio indicated by the multimeter and the ratio of the reference voltages applied to the meter inputs. A sufficient number of repetitions are conducted until the meter calibration can be calculated with a standard uncertainty of about 0.0003 %.

The measurements also establish the linearity of the multimeter, represented by the uniformity of the calibration factor over the range from 0.5 mV/V to 10 mV/V. The multimeters used in the NIST force laboratory demonstrate a linearity sufficient to enable a single meter calibration factor to be applied; the uncertainty associated with nonlinearity is about 0.0001 %.

**Fig. 3:**
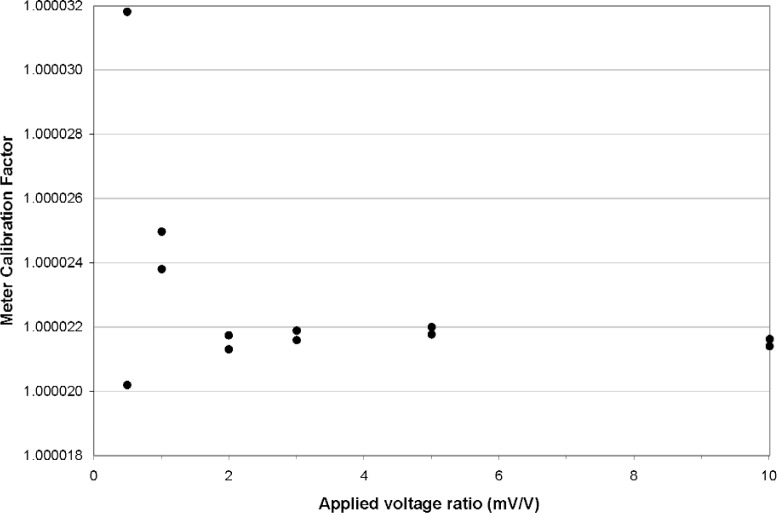
Plot of a typical multimeter calibration in voltage-ratio mode by the NIST Quantum Electrical Metrology Division.

The results of a typical calibration by the Quantum Electrical Metrology Division is shown in Fig. 3, plotted as the voltage-ratio indicated by the meter divided by the ratio of the reference voltages.

The meter calibration factors for the eight computing multimeters used by the force laboratory range from about 0.999985 to 1.000070. The standard uncertainty in the load cell response *R* that is associated with the NIST Quantum Electrical Metrology Division determination of these calibration factors is

$$u_{\text{va}}=0.000003\;R.$$ (21)

The standard uncertainty associated with the multimeter linearity is

$$u_{\text{vb}}=0.000001\;R.$$ (22)

### 4.2 Intercomparison of NIST Force Laboratory Instruments

The NIST Mass and Force Group maintains eight identical 8½ digit computing multimeters for voltage-ratio measurements at six deadweight machines, ensuring that sufficient multimeters are available to accommodate load cells with multiple strain gauge bridge networks. While one of these multimeters is selected at least yearly for a primary calibration by the NIST Quantum Electrical Metrology Division, a procedure is necessary for frequent checks of the calibration of all of the multimeters. The method currently employed for this purpose makes use of a precision load cell simulator to serve as a “voltage-ratio transfer standard”; this device is used to transfer the primary calibration by the Quantum Electrical Metrology Division to the other seven multimeters.

The load cell simulator is a passive electrical network with connections and impedances representative of most load cells and an output providing a voltage-ratio that is selectable in steps from 0 mV/V to 10 mV/V. It is stable within ±0.000005 mV/V over a 24 h period. Each multimeter is connected, one at a time, to the load cell simulator output terminals and readings are taken in voltage-ratio mode over a sampling time interval of 150 s. For this sampling time the standard deviation of repeated measurements is ≤0.000003 mV/V. Readings are taken for simulator output settings of 10 mV/V, 2.5 mV/V, and 0 mV/V, and for each of two excitation conditions: +10 VDC and −10 VDC. These measurements are completed for all of the multimeters within a half day’s time.

The multimeter which is most recently calibrated by the Quantum Electrical Metrology Division is used to determine the output of the simulator at the relevant voltage-ratio settings. This simulator output is then used to calculate a meter calibration factor for each of the other seven multimeters. This factor is calculated separately for each ratio setting from the +10 V and −10 V excitation values and again from the +10 V and 0 V excitation values.

The results give a check on each meter’s linearity as well as its proper functioning at both positive and negative excitation voltage polarity. The calibration factor for each multimeter is determined with a relative standard uncertainty of 0.0003 % to 0.0004 %. Since this factor is a multiplier to all load cell response readings *R* acquired for subsequent force calibration measurements, the standard uncertainty in *R* that is associated with the comparison calibrations of the multimeters with the simulator is

$$u_{vc}\approx 0.000004\;R.$$ (23)

**Fig. 4:**
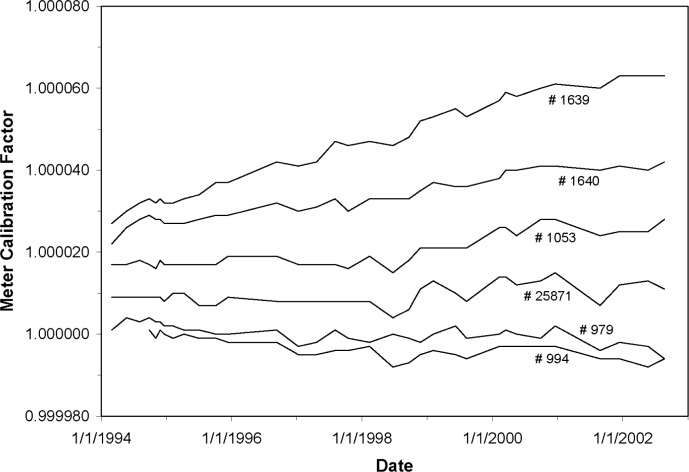
Plots of the repeatability of the calibration factors for six of the multimeters over an 8 year time period. The numbers shown are the instrument serial numbers.

Figure 4 shows plots of the repeatability of the meter calibration factors for six of the multimeters over an 8 year time period. The factors were determined from the procedures described above. The multimeters are identified on the plot by serial number. Six different meters, some repeatedly, were calibrated at intervals by the Quantum Electrical Metrology Division for use as references during this period.

The plots shown in Fig. 4 indicate how precisely the calibrations of the multimeters can be maintained by the established procedures. If linear least-squares computations are performed for the data for the multimeters shown here, the standard deviation of the individual data points about the fitted line may be calculated for each multimeter. These standard deviations range from 0.000002 to 0.000004 for the multimeters shown. These results demonstrate that while the uncorrected readings from different multimeters may vary by 0.0075 %, appropriate calibration procedures can maintain agreement among all of these units to within 0.0005 %

If the actual factor for the multimeter being used as a reference for the other seven multimeters should begin to drift after its Quantum Electrical Metrology Division calibration, the comparison calibrations would show this drift as a simultaneous change in the factor for the other seven meters. Since an actual similar change in the response of seven meters is statistically unlikely, the calibration procedures described above have some inherent safeguards against undetected data corruption resulting from a single malfunctioning instrument.

### 4.3 Standard Uncertainty in Voltage-Ratio Instrument Calibration

The standard uncertainty, *u*_v_, in the calibration of the NIST voltage-ratio instrumentation, incorporating all significant uncertainty components, may now be calculated from

$${u_{v}}^{2}={u_{\text{va}}}^{2}+{u_{\text{vb}}}^{2}+{u_{\text{vc}}}^{2},$$ (24)

where *u*_va_, *u*_vb_, and *u*_vc_ are given by Eqs. (21), (22), and (23), respectively. For the NIST instrumentation, this calculation yields

$$u_{v}=0.000005\;R.$$ (25)

## 5. Deviations of Measurement Data from the Least-Squares Fit

A force calibration provides the transducer response as a function of applied force in the form of Eq. (1) by deriving the coefficients *A_i_* from a least-squares fit to the calibration data. The uncertainty associated with the variation in the measured data from the fitted curve is represented by the standard deviation *u*_r_ in Eq. (2). This standard deviation is calculated according to ASTM E 74-04 from

$${u_{r}}^{2}=\left(\sum {d_{j}}^{2}\right)/\left(n-m\right),$$ (26)

where the *d_j_* are the differences between the measured responses, *R_j_*, and the responses calculated from Eq. (1)*n* is the number of individual measurements in the calibration data set, and *m* is the order of the polynomial plus one.

Many factors contribute to the standard deviation *u*_r_, including (a) random errors associated with the resolution, instrument noise, and repeatability of the indicator; (b) variations caused by swinging of the weights; (c) deviations of the assumed transducer response modeled by Eq. (1) and the true transducer response; (d) irregularities due to the characteristics of the transducer being calibrated, such as creep, hysteresis, and sensitivity to placement in the force machine. Some of these factors can be minimized by procedural technique, such as choosing optimum indicator sampling parameters, achieving precise transducer alignment, maintaining of machine weight changing and motion inhibiting mechanisms, and properly selecting the order of fit for the least-squares analysis.

The transducer related effects usually make up the largest share of the deviations incorporated into *u*_r_ and constitute the dominant contributors of overall measurement uncertainty. Usual calibration practice enables these effects to be quantified by limiting the number of forces that are applied before returning to zero force and by repeating the measurement sequence for several reorientations of the transducer in the force machine. The dependence of the load cell response upon previously applied forces and upon the degree of misalignment of the applied force relative to the load cell axis then becomes evident, both in the quantity *u*_r_ and in a plot of the deviations from the fitted curve. A detailed discussion of these uncertainty sources is given in C. P. Reeve [19].

**Fig. 5:**
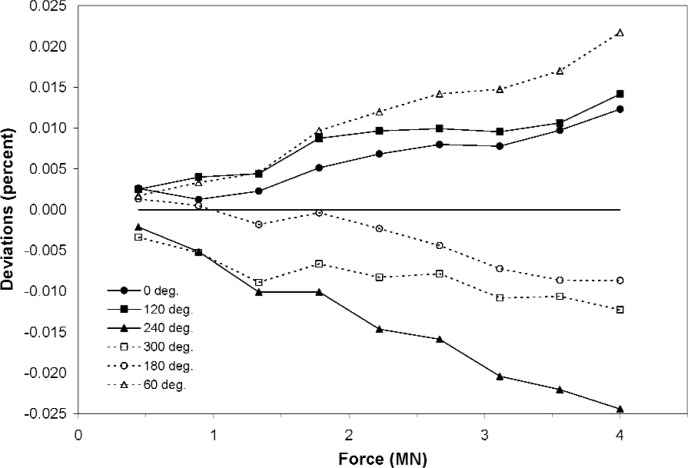
Deviations of individual data from a least-squares fit for a load cell with relatively high sensitivity to orientation within the NIST 4.448 MN deadweight machine.

An example of a load cell with a relatively high sensitivity to angular position with respect to the NIST 4.448 MN deadweight machine loading platens is shown in Fig. 5. A measurement sequence consisting of nine forces was conducted for each of six orientations. The ordinates represent the deviations in the data about a least-squares fit in the form of a third-order polynomial. The fitted curve is represented on this plot as a horizontal line of zero deviation. The standard deviation *u*_r_ of these data about the fitted curve is 0.011 % of the load cell response at maximum force. The combined standard uncertainty *u*_c_, calculated from Eqs. (2), (20), and (25), is 0.011 % of the response at maximum force; the expanded uncertainty, for a coverage factor, *k*, of 2, is thus 0.022 %.

**Fig. 6:**
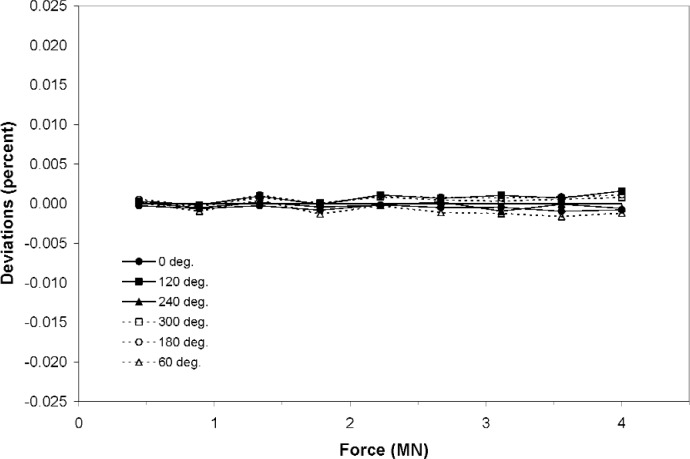
Deviations of individual data from a least-squares fit for a load cell with very low sensitivity to orientation within the NIST 4.448 MN deadweight machine.

An example of a load cell with a very low sensitivity to orientation within the NIST 4.448 MN deadweight machine is shown in Fig. 6. All measurement parameters were identical to the parameters used in the measurement depicted in Fig. 5. The scales of the axes are the same in the two plots. The standard deviation *u*_r_ of the data of Fig. 6 about the fitted curve is 0.0008 % of the load cell response at maximum force. The combined standard uncertainty *u*_c_, calculated from Eqs. (2), (20), and (25), is 0.0011 % of the response at maximum force. The relative expanded uncertainty is thus 0.0022 %.

For force calibrations that have been performed at NIST, the lower end of the uncertainty range associated with load cell characteristics is represented by a value of *u*_r_ of 0.0003 % of the response at maximum force, when *u*_r_ is calculated from Eq. (26) using data from at least three orientations within the deadweight machine. The corresponding expanded uncertainty is 0.0015 % for calibrations performed with NIST’s voltage-ratio instrumentation. If the load cell is paired with a dedicated indicator, thus eliminating the component *u*_v_ of Eq. (24), the relative expanded uncertainty is 0.0012 %.

It is not practical to define the upper end of the uncertainty range, since this represents force transducers of less precise design or with certain problems that may be correctable. Occasionally a transducer calibration yields a value of *u*_r_ in excess of 0.1 %. Depending on the application, such a calibration may still be valuable to the customer.

The plots in Figs. 5 and 6 demonstrate that the dependence of the measurement uncertainty upon the transducer characteristics prevent predetermination of the final measurement uncertainty.

## 6. Conclusions

The previous sections have explained the components of measurement uncertainty in NIST calibrations of force transducers. The standard uncertainty in the transducer response *R* due to the uncertainties in the forces applied by the NIST deadweight force standards is given by Eq. (20) as *u*_f_ = 0.000005 *R*. The standard uncertainty in *R* due to the uncertainty in the calibration of the NIST voltage-ratio instrumentation is given by Eq. (25) as *u*_v_ = 0.000005 *R*. The standard deviation *u*_r_ of the measured transducer responses relative to the fitted curve derived from the calibration data is dependent upon the characteristics of the transducer being calibrated; its value may range from 0.0003 % to more than 0.1 % of the response at rated capacity. The final expanded uncertainty is *U* = 2*u*_c_, where the combined standard uncertainty is calculated according to Eq. (2) as *u*_c_=(*u*_f_^2^ + *u*_v_^2^ + *u*_r_^2^)^1/2^. The relative values for *U* may range from 0.0012 % for exceptionally precise transducers to more than 0.2 %.

Certain aspects of the force calibration analysis and the evaluation of uncertainty are under study for possible refinement, such as may be appropriate as force transducer technology improves. The refinements being considered at the force laboratory include (a) revisions to the least-squares algorithm employed for deriving the polynomial coefficients of Eq. (1) in order for the fitting computation to take adequate account of the uncertainty in applied force; (b) incorporation of an additional error term, if necessary, when the order of fit requested by the customer differs from the mathematically best possible order of fit; and (c) expression of the expanded uncertainty in the transducer response as a function of the response over the range of the transducer, rather than as a single number that is understood to represent the uncertainty for points distributed randomly throughout the range.

The values of uncertainty reported here are maintained through a quality assurance program followed by the NIST Mass and Force Group staff. This program includes diligent mechanical inspection and maintenance of the deadweights and associated loading mechanisms. The program also includes maintenance of the calibration of the NIST voltage-ratio instrumentation, carried out through secondary calibration of the computing multimeters as described in Sec. 4.2 on a quarterly basis, and primary calibration of a multimeter by the NIST Quantum Electrical Metrology Division at least yearly. In addition, intercomparisons of NIST’s deadweight machines are conducted through the use of a set of precision force transducers as transfer standards among the machines. While it is recognized that the uncertainty in the response of the force transducers is greater than the uncertainty in the applied force, this process is useful in maintaining assurance that detectable faults have not appeared in one or more segments of the measurement system.
